# Maternal HLA Ib Polymorphisms in Pregnancy Allo-Immunization

**DOI:** 10.3389/fimmu.2021.657217

**Published:** 2021-03-30

**Authors:** Gry Persson, Christophe Picard, Gregory Marin, Cecilie Isgaard, Christina Seefeldt Stæhr, Nicolas Molinari, Jacques Chiaroni, Morten Lebech, Thomas Vauvert F. Hviid, Julie Di Cristofaro

**Affiliations:** ^1^ Centre for Immune Regulation and Reproductive Immunology (CIRRI), Department of Clinical Biochemistry, The ReproHealth Research Consortium ZUH, Zealand University Hospital, Roskilde, Denmark; ^2^ Aix Marseille Univ, CNRS, EFS, ADES, “Biologie des Groupes Sanguins”, Marseille, France; ^3^ Immunogenetics Laboratory, Etablissement français du Sang PACA Corse, Marseille, France; ^4^ Unité de Recherche Clinique, Biostatistique et Epidémiologie, Département de l’Information Médicale (DIM) Hôpital La Colombière, Montpellier, France; ^5^ Department of Clinical Medicine, University of Copenhagen, Copenhagen, Denmark; ^6^ Department of Obstetrics and Gynecology, Zealand University Hospital, Roskilde, Denmark

**Keywords:** HLA class Ib, anti-HLA alloimmunization, IgG4, tolerance, pregnancy

## Abstract

During pregnancy the formation of alloreactive anti-human leukocyte antigen (HLA) antibodies are a major cause of acute rejection in organ transplantation and of adverse effects in blood transfusion. The purpose of the study was to identify maternal HLA class Ib genetic factors associated with anti-HLA allo-immunization in pregnancy and the degree of tolerance estimated by IgG4 expression. In total, 86 primiparous women with singleton pregnancies were included in the study. Maternal blood samples and umbilical cord samples were collected at delivery. Clinical data were obtained. Maternal blood serum was screened for HLA class I and II antibodies, identification of Donor Specific Antibody (DSA), activation of complement measured by C1q and IgG4 concentrations. Mothers were genotyped for HLA class Ib (*HLA-E, -F* and *-G*). Anti-HLA class I and II antibodies were identified in 24% of the women. The maternal HLA-E*01:06 allele was significantly associated with a higher fraction of anti-HLA I immunization (20.0% *vs.* 4.8%, p = 0.048). The maternal HLA-G 3’-untranslated region UTR4-HLA-G*01:01:01:05 haplotype and the HLA-F*01:03:01 allele were significantly associated with a low anti-HLA I C1q activation (16.7% *vs*. 57.1%, p = 0.028; 16.7% *vs.* 50.0%, p = 0.046; respectively). Both *HLA‑G * and *HLA*-F*01:03:01 showed significantly higher levels of IgG4 compared with the other haplotypes. The results support an association of certain *HLA class Ib* alleles with allo-immunization during pregnancy. Further studies are needed to elucidate the roles of HLA-E*01:06, HLA-F*01:03 and *HLA‑G UTR4* in reducing the risk for allo-immunization.

## Introduction

Alloreactive anti-human leukocyte antigen (HLA) antibodies formed during pregnancy are a major cause of acute rejection in organ transplantation and of adverse effects in blood transfusion, such as febrile non-hemolytic transfusion reactions, immunological platelet refractoriness or transfusion-related acute lung injury (TRALI) (1, 2).

Sir Peter Medawar was among the first to recognize the immunological paradox of pregnancy. The current study is in line with this important scientific theme by investigating a role of maternal *HLA class Ib* polymorphisms in pregnancy allo-immunization.

Studies on pregnancy-induced alloimmunization have shown that 18 to 74% of women with a history of pregnancy have anti-HLA alloantibodies. The discrepancy between studies may be due to screening differences, both in method sensitivity and the sampling time after delivery, as HLA antibodies may persist or become gradually undetectable over time (1–5). The level of anti-HLA antibodies increases with the number of pregnancies and the number of children delivered (1–5). Biological and genetic factors have been associated with pregnancy-induced anti-HLA alloimmunization, however, the process remains poorly understood.

In pregnancy, immunization seems to be driven by special mechanisms. It is hypothesized that among decidual antigen presenting cell (APC) populations (classical macrophages, classical mature dendritic cells (DC), immature DC and myeloid DC), specialized APCs present fetal antigens to the maternal immune system. However, the exact mechanisms that induce a state of tolerance to fetal antigens are unclear (6). Various immunoglobulins (Igs) can play an important role in regulating the pregnancy process and maintaining tolerance to the fetus (7). The average relative levels of IgGs and IgMs in placentas are slightly higher and lower, respectively, than in healthy individuals (7). Considering IgGs, Lowe et al. (2013) suggested that the distribution of anti-HLA IgG subclasses (IgG1-IgG4) varied according to the sensitizing event, i.e. graft, transfusion or pregnancy (8, 9). IgG4, whose altered Fc domain reduces antibody-dependent cell-mediated cytotoxicity, complement-dependent cytotoxicity, clearance of antigens and phagocytosis (10), may protect the fetus that harbors paternal antigens from the mother’s effector immune mechanisms by competing for antigen and Fc binding with the other IgG subclasses (11, 12). The relevance of IgG4 as an immune tolerance marker is, however, challenged in a transplant context. Lowe et al. found that HLA-DQ specificity and IgG4 were associated with chronic kidney graft failure (8), whereas Lobashevsky et al. reported that IgG2/IgG4 donor specific antibodies (DSA) were associated with antibody-mediated rejection (AMR)-free transplants (13).

Other molecules such as the HLA Ib molecules (HLA-G, HLA-E and HLA-F) also play a major role in inducing tolerance during pregnancy (14). HLA-G, HLA-E and HLA-F are expressed at the materno-fetal interface, whereas trophoblast lacks all other classical class I antigens except HLA-C (15, 16). Their tolerogenic properties confer a peculiar interest in comprehension of the alloimmunization process during pregnancy.

HLA-G interacts with the inhibitory receptors Ig-like transcript 2 (ILT2), ILT4 and the killer cell immunoglobulin-like receptor (KIR) 2DL4, expressed by a wide range of immune cells including monocytes, T cells, B cells and NK cells (17–20). Reduced HLA-G expression is associated with pregnancy complications (21). Different HLA-G alleles have been associated both to the HLA-G expression level (22–26) and a better tolerance in pregnancy (27–30). A previous study on immunization in pregnancy suggested that the HLA-G*01:01:01:04 allele is protective against anti-HLA class II immunization (31).

HLA-E regulates NK cell function through the C-type lectin receptor CD94 combined NKG2 sub-units expressed on NK and CD8+ αβ T cells (32, 33). HLA-E is expressed by placental and trophoblast cells but also by immune effectors (34). HLA-E mRNA expression is similar in peripheral blood mononuclear cells (PBMCs) from homozygous HLA-E*01:01 or HLA-E*01:03 individuals (35). Conversely, HLA-E*01:03 membrane-bound expression is higher when compared to HLA-E*01:01; furthermore, HLA‑E membrane-bound expression depends on the affinity for HLA Ia peptides and appears to be related to cell activation (34). Increased frequency of homozygosity for HLA-E*01:01 in Egyptian women with recurrent miscarriage was reported (15).

HLA-F participates in immune regulation in pregnancy, infections, autoimmunity and cancer especially through the KIR3DS1 receptor (16, 36–38). Like HLA-E, HLA-F membrane-bound expression is associated with a state of activation (39). Little data are available concerning associations between specific *HLA-F* alleles and the expression of HLA-F or clinical outcome. HLA-F*01:01:02 was associated with higher mRNA expression (40), and HLA-F*01:03 was associated with lower Hepatitis B virus DNA level in Tunisian patients with hepatitis B virus chronic infection (41).

The main goal of the current study was to identify maternal *HLA Ib* genetic factors associated with anti-HLA allo-immunization in pregnancy and the level of tolerance estimated by IgG4 expression. To circumvent problems with the aspect of multigravity immunization, this study includes primaparous women with no history of pregnancy complications or blood transfusion.

## Material and Methods

### Patients, Data and Sample Collection

The study was designed together with the Centre for Immune Regulation and Reproductive Immunology (Zealand University Hospital and the University of Copenhagen, Denmark). The recruitment of the pregnant women was completed during their last visit at the birth clinic at the Department of Obstetrics and Gynaecology, Zealand University Hospital, Roskilde, Denmark. All subjects gave written informed consent for participation in the study prior to sample collection. The protocol has been approved by the local ethics committee for Region Zealand (National Committee on Health Research Ethics; No. SJ591).

Eighty-six women were included in the study. Maternal blood samples and umbilical cord samples were collected at delivery. Collected data included maternal age, fetal gender, pregnancy and transfusion history. All included women were primigravidae and had singleton pregnancies. None of the women had any recorded previous or present interruption of pregnancy or dead infant. No women experienced pregnancy complications or showed any history of transfusion.

Whole blood collected in serum tubes (BD, #367624) was left to coagulate at room temperature (RT) and maternal serum was obtained by centrifugation (RT, 2000 x g, 10 min). Serum was stored at ‑80°C. Genomic DNA was extracted from EDTA blood (BD, # 365900) of the mother and cord using the QIAsymphony DSP DNA Midi Kit for 1000μl samples using a QIASymphony instrument (QIAGEN, Hilden, Germany). Genomic DNA concentration was measured using DropSense 96 and DropQuant (Trinean, Gentbrugge, Belgium) before being stored at ‑20°C. All samples were shipped on dry ice to Etablissement Français du Sang PACA Corse, Marseille, France, for further analysis.

### Maternal Anti-HLA Alloimunization

Maternal serum from whole blood collected at delivery was screened for detection of HLA class I and class II antibodies, identification of Donor Specific Antibody (DSA) and the ability of antibody to bind and activate complement as measured by C1q. Sera were screened for anti-HLA antibodies with Luminex LABScreen Mixed (SPA-Screen Solid phase screen, One Lambda Inc, West Hills, CA, USA). Sera that were positive in class I and class II SPA-Screen assays (normalized background (NBG) ratio > 3 for any class I multi-antigen bead and NBG ratio > 4.5 for any class II multi-antigen bead) was further tested to identify antibody specificity by LABScreen Single Antigen (SAB, One Lambda Inc, West Hills, CA, USA) according to the manufacturer’s instructions. Sera positive in SAB (MFI > 500) were further tested to identify complement-fixing DSA using a C1q Screen assay (One Lambda Inc, West Hills, CA, USA) according to the manufacturer’s instructions (MFI > 1,000). HLA FUSION 2.0 software (One Lambda) on the LABScan100 flow cytometer (Luminex Inc.) was used for interpretation.

Identification of mismatched inherited paternal HLA antigens (IPA) was based on two-field resolution HLA typing of both mother and child. The *HLA-A*, *-B*, *-C*, *-DRB1* and *-DQB1* genes from both maternal and child genomic DNA were sequenced by Next Generation Sequencing (NGS) using a 5 loci kit V2 (H23 holotype HLA 96/5 config A&CE V2 from Omixon Biocomputing KFT, Budapest, Hungary).

### Maternal IgG4 Dosage

Measurement of IgG4 was performed in duplicate on maternal serum samples using the ELISA for quantitative detection of human IgG4 (#BMS2095, Life Technologies, Waltham, Massachusetts, U.S.) according to the manufacturer’s protocol. Detection threshold was 10 IU/mL.

### Maternal *HLA Ib* Typing

Mothers were typed for *HLA-E*, *-F* and *-G*. Furthermore, the *HLA-G* haplotype based on the regulatory regions was also sequenced. The *HLA Ib* genes were sequenced by NGS (42–44). The *HLA-E* gene was sequenced from position -257 to +3072 (primer sequences were TCATCTCTGTGGGCTACGTG and TCAGACCCCCAGAATCTCAC); the *HLA-F* gene was sequenced from position -266 to +3249 (GTGGCTCTCAAGGGCTCAG and GCAACAACCAAAGCATCGTA); the *HLA-G* gene was sequenced from position -1983 to +3447 (primer sequences: AGGAGCTGACACAGGAGGAA and CAGCTGAGCAGTGACCACAT). Amplification was performed using the Long Range PCR Kit (Qiagen). PCR fragments were sequenced using an NGS platform (MiSeq, Illumina, San Diego, California, U.S.).

*HLA-E, -F* and -*G* NGS data were analyzed through PolyPheMe software (Xegen, Gemenos, France) based on the HLA sequences listed in the official IMGT/HLA database (45). High-resolution typing of *HLA-G* regulatory regions (H1 to H74) was performed according to all polymorphic variations from position -1983 to -1 in the 5’UTR and from +2540 to +3447 in the 3’UTR. The *HLA-G* haplotype identification numbers were coded as previously described (44, 46). Allelic, UTR and haplotype frequencies were estimated using PHASE and an EM algorithm implemented in the Gene[Rate] computer tools (47).

### Data Interpretation and Statistical Analysis

Quantitative data are given in median and range [min-max] or mean and standard deviation (SD). Associations between anti-HLA antibody, DSA, C1q activation, IgG4 expression and *HLA Ib* genetic polymorphism were tested with logistic regression, when the predicted variable was categorical, and general linear regression when the predicted variable was quantitative.

Univariate analyses were performed on each of the following variables or features: anti-HLA antibody, DSA, C1q activation according to *HLA Ib* genetic polymorphisms and IgG4 expression. The False Discovery Rate correction for multiplicity was applied; no power analysis was performed. Variables with corrected p-values below 0.15 in univariate analysis were further investigated in multivariate analysis. Results were considered significant if p-values were below 0.05 after stepwise type selection.

Analysis of IgG4 expression according to *HLA Ib* genetic polymorphisms was performed by Wilcoxon-Mann-Whitney test. Results were considered significant if p-values were below 0.05.

Results of association analyses with quantitative variables were expressed as estimates and standard errors (Std error), whereas results of association analyses with qualitative variables were expressed as odd ratios (OR) and 95% confidence intervals (CI 95%).

## Results

### Population Characteristics

The median age of the mothers was 28 years (range 18-40 years), median gestational age was 281 days (range 236-299 days) and median birth weight was 3638 g (range 1730-5030 g).

Twenty-one women (24%) had anti-HLA class I antibodies, 21 women (24%) had anti-class II antibodies; 10 (11%) women had both. Fifteen of the women (17%) had anti-HLA class I antibodies that activated complement, and 4 (5%) women had anti-HLA class II antibodies that activated complement (**Table 1**). Women with anti-HLA antibodies directed against IPA, defined as donor Specific Antibodies (DSA), were considered for analysis (21% for anti-class I antibodies and 21% for anti-class II antibodies). All women but two had measurable IgG4 in their serum (mean: 870 IU/mL; range: 0-3450 IU/mL). The results are presented in **Table 1**.

**Table 1:** Characteristics of participants and sera.

Number of Women	86
Age (years) (median and range)	28 [18-40]
Gestational age (days) (median and range)	281 [236-299]
Birth weight (gram) (median and range)	3638 [1730-5030]
Fetal gender (n Female/Male)	35/51
HLA class I antibody (n) (percentage positive)	21 (24%)
HLA class II antibody (n) (percentage positive)	21 (24%)
HLA class I and class II antibody (n) (percentage positive)	10 (11%)
DSA HLA class I antibody (n) (percentage positive)	18 (21%)
DSA HLA class II antibody (n) (percentage positive)	18 (21%)
HLA class I antibody with complement activation (n) (percentage positive)	15 (17%)
HLA class II antibody with complement activation (n) (percentage positive)	4 (5%)
IgG4 (IU/ml) (mean and SD)	895 (± 829)

### Maternal E*01:06 Allele Association With a Higher Frequency of Anti-HLA I Immunization

The HLA-E*01:06 allele was associated with anti-HLA I antibody in multivariate analysis (20% *vs.* 4.8%; p = 0.048; OR = 5.0, CI [1.0-24.6]; **Table 2**). *HLA Ib* polymorphisms and IgG4 with corrected p-values below 0.15 in univariate analyses are shown in **Table 2**.

**Table 2:** HLA class I antibody according to *HLA Ib* polymorphisms. All samples were included in the analysis, missing genetic data led to the exclusion of the concerned sample from analysis.

	Presence	Absence	OR (95% CI) (^∆^Univariable; ^†^Multivariable)
E*01:06 (8.4%; n=7/83)	20.0% (n=4/20)	4.8% (n=3/63)	5.0 (1.0-24.6); p = 0.048^†^
*HLA-G H02* (15.1%; n=13/86)	28.6% (n=6/21)	10.8% (n=7/65)	3.3 (1.0-11.3); p = 0.056^∆^
*HLA-G H04* (18.6%; n=16/86)	4.8% (n=1/21)	23.1% (n=15/65)	0.2 (0.1-1.3); p = 0.093^∆^
*HLA-G H21* (3.5%; n=3/86)	9.5% (n=2/21)	1.5% (n=1/65)	6.7 (0.6-78.4); p = 0.128^∆^
IgG4 (IU/mL)	592.7 ( ± 583.1)	986.7 ( ± 823.2)	0.9 (0.8-1.0); p = 0.07^∆^

No multiple imputation was used (Logistic Regression). The HLA-G haplotype identification numbers (HLA-G H02, H04, H21) were coded as previously described (44, 46). HLA-G H02 and H04 are associated with UTR4 and HLA-G H21 is associated with UTR5.

### Maternal HLA-G UTR4-G*01:01:01:05 Haplotype and F*01:03:01 Allele Association With Lower Frequency of Anti-HLA I C1q Activation and Higher IgG4 Expression

The *HLA-G UTR4* haplotype, in linkage disequilibrium (LD) with G*01:01:01:05 (44), and the *HLA-F* allele F*01:03:01 were both associated with absence of HLA class I antibody complement activation (respectively p = 0.028; OR = 0.1, CI [0.01-0.8]; and p = 0.046; OR = 0.1, CI [0.01-0.97]; **Table 3**) and higher IgG4 expression (Wilcoxon-Mann-Whitney, respectively p = 0.03, 1200.2 ( ± 975.9) IU/mL *vs.* 671.5 ( ± 552.7) IU/mL; and p = 0.05, 1165.6 ( ± 959.5) IU/mL *vs*. 681.8 ( ± 549.4) IU/mL, **Table 4**).

**Table 3:** HLA I antibody complement activation according to *HLA Ib* polymorphisms.

	Activation	No activation	OR (95% CI) (^∆^Univariable; ^†^Multivariable)
F*01:03:01 27.8% (n=5/18)	16.7% (n=2/12)	50.0% (n=3/6)	0.1 (0.01-0.97); p = 0.046^∆^
*HLA-G UTR4* 31.6% (n=6/19)	16.7% (n=2/12)	57.1% (n=4/7)	0.1 (0.01-0.8); p = 0.028^†^

Samples with a HLA I antibody were included in the analysis (n=21), missing genetic data led to the exclusion of the concerned sample from analysis. No multiple imputation was used (Logistic Regression).

**Table 4:** IgG4 expression (Mean ( ± SD), IU/mL) according to *HLA Ib* genetic polymorphisms (Wilcoxon-Mann-Whitney test).

	Presence	Absence	P-value
*HLA-G UTR4*	1200.2 ( ± 975.9)	671.5 ( ± 552.7)	0.03
F*01:03:01	1165.6 ( ± 959.5)	681.8 ( ± 549.4)	0.05

## Discussion

Peter Medawar’s pregnancy paradox from 1953 was the first to propose an explanation for maternal acceptance of the semi-allogenic fetus during pregnancy. Although much knowledge has been added to the field since then, pregnancy remains a model to study the development of allogenic tolerance and anti-HLA alloantibodies. Anti-HLA alloantibodies induced by pregnancy are of importance because of clinical adverse effects of pre-transplantation donor-specific antibodies (DSA) in transfusion and in the graft. Their prevalence is reported with wide ranges probably because of technical differences. When anti-HLA alloantibodies may become undetectable, additional markers of alloimmunization would help to prevent clinical adverse effects.

The expression of HLA class Ib molecules is well described for the establishment of immune tolerance in pregnancy (22, 48). HLA-G and HLA-E expression are described to be associated with genetic polymorphisms, whereas little data are available regarding HLA-F (14, 38).

We aimed to identify maternal *HLA Ib* genetic markers for allo-immunization during pregnancy and the level of tolerance estimated by IgG4 expression. IgG4 expression may be associated with IPA tolerance in pregnancy, possibly by competing with fetus antigens without maternal immune cell activation (8, 11, 12). IgG4 antibody subclass may be an illustration of how the maternal immune systems promote tolerance to foreign paternal antigens.

The current study included 86 primigravidae women for which genomic DNA, sera and cord blood were available. Sera were collected at delivery. Maternal anti-HLA antibodies were screened and their ability to activate complement was analyzed. Mothers were genotyped for *HLA-G, HLA-E* and *HLA-F*, as well as analyzed for the *HLA-G* regulatory region haplotype. IgG4 concentrations were measured in the maternal sera.

Anti-HLA class I and class II antibodies were identified in 24% of the women, respectively, of which 17% and 5% presented anti-HLA class I and class II antibodies that activated complement.

We showed that maternal E*01:06 allele was associated with a higher percentage of anti-HLA class I immunization in multivariate analysis. This allele is virtually present only in Europeans and Europeans descents and could not be explored for its expression or association with clinical issues. HLA-E*01:06 displays HLA-E*01:03 specific variation and a non-synonymous mutation at position +1857 (49, 50).

We found that the maternal *HLA-G UTR4* haplotype and the F*01:03:01 allele were both associated with lower anti-HLA I complement activation and higher IgG4 expression. Both results are consistent with and support that both alleles are associated with harmless antibodies (i.e. absence of C1q activation and IgG4 subclass) and with an immune response maintained under control. *HLA*-*G UTR4* was associated with a shorter time-to-pregnancy in couples undergoing infertility treatment (51) and was found more frequent in healthy individuals than in asthmatic patients (44). Concerning HLA-F, in silico analysis reported equivalent mRNA expression in F*01:03 and F*01:01:01 (40). However, HLA-F expression seems to be driven also by cell interaction and activation. Although HLA-F*01:03 displays a polymorphism (p.251S>P) outside the peptide groove, Ho et al. showed that F*01:03 binds and presents different peptides than F*01:01 and F*01:02 (52). Biological consequences of this feature remain to be explored.

No *HLA Ib* genetic polymorphism or IgG4 expression was significantly associated with anti-HLA II immunization, suggesting that anti-HLA I and anti-HLA II immunization may be driven by different factors, as previously suggested (31). We could not investigate previous published data on the protective role of H03-UTR6-HLA-G*01:01:01:04 on anti-HLA II immunization during pregnancy (31) as this haplotype was not observed in the cohort under study.

In conclusion, our results support an association of *HLA Ib* alleles with allo-immunization during pregnancy. Moreover, this study suggests that specific *HLA Ib* alleles are associated with different anti HLA class I antibody properties. HLA Ib molecules are ligands for immune cell inhibitory receptors. B cells require a co-stimulatory signal for maturation, affinity maturation and proper class switching. ILT2 expressed by B cells has high affinity for HLA-G. Interaction of ILT2^+^ B cells with HLA-G has been shown to inhibit their proliferation, antibody production, dampen migration of germinal center B cells as well promoting a shift from Th1 to Th2 response by reducing secreted IFN-γ and IL-2, while increasing IL-4 and IL-10 production (53). Interestingly, a xenograft mice model injected with HLA-G^+^ xenogeneic cells had reduced levels of anti-xenograft antibodies (53). Regulatory B cells are characterized by IL-10 production, and IgG4 expression has been shown to be confined to this subset of B cells (54), thus specific *HLA Ib* alleles, associated to differential quantitative or qualitative expression, may modify these ligand-receptor interactions and the course of immune cells response to foreign antigens.

Association of an allo-immunization directed against the fetus without activation of immune effector-cells would be in line with the concept of acquired immunological tolerance in a pregnancy context.

## Data Availability Statement

The raw data supporting the conclusions of this article will be made available by the authors, without undue reservation.

## Ethics Statement

The studies involving human participants were reviewed and approved by local ethics committee for Region Zealand, National Committee on Health Research Ethics; No. SJ591. The patients/participants provided their written informed consent to participate in this study.

## Author Contributions

All authors listed have made a substantial, direct, and intellectual contribution to the work and approved it for publication.

## Funding

This study was funded by Etablissement Français du Sang (APR16) and by the ReproHealth Research Consortium Zealand University Hospital.

## Conflict of Interest

The authors declare that the research was conducted in the absence of any commercial or financial relationships that could be construed as a potential conflict of interest.
